# Mitochondrial Health Through Nicotinamide Riboside and Berberine: Shared Pathways and Therapeutic Potential

**DOI:** 10.3390/ijms27010485

**Published:** 2026-01-02

**Authors:** Federico Visalli, Matteo Capobianco, Francesco Cappellani, Lorenzo Rapisarda, Alfonso Spinello, Alessandro Avitabile, Ludovica Cannizzaro, Caterina Gagliano, Marco Zeppieri

**Affiliations:** 1 Department of Ophthalmology, University of Catania, 95123 Catania, Italy; federico.visalli13@gmail.com (F.V.);; 2 Department of Medicine and Surgery, University of Enna “Kore”, 94100 Enna, Italy; 3 Mediterranean Foundation “G.B. Morgagni”, 95125 Catania, Italy; 4 Azienda Sanitaria Provinciale 8 Siracusa, Presidio Ospedaliero Umberto I, 96100 Siracusa, Italy; 5 Faculty of Medicine, University of Catania, 95123 Catania, Italy; 6 Department of Ophthalmology, University Hospital of Udine, 33100 Udine, Italy; 7 Department of Medicine, Surgery and Health Sciences, University of Trieste, 34127 Trieste, Italy

**Keywords:** nicotinamide riboside, nicotinamide, berberine, NAD^+^ metabolism, mitochondrial dysfunction, retinal ganglion cells, neuroprotection, oxidative stress, cardiometabolic disease

## Abstract

Mitochondrial dysfunction represents a central hallmark of aging and a broad spectrum of chronic diseases, ranging from metabolic to neurodegenerative and ocular disorders. Nicotinamide riboside (NR), a vitamin B_3_ derivative and efficient precursor of NAD^+^ (nicotinamide adenine dinucleotide), and berberine (BBR), an isoquinoline alkaloid widely investigated in metabolic regulation, have independently emerged as promising mitochondrial modulators. NR enhances cellular NAD^+^ pools, thereby activating sirtuin-dependent pathways, stimulating PGC-1α–mediated mitochondrial biogenesis, and triggering the mitochondrial unfolded protein response (UPR^mt^). BBR, by contrast, primarily activates AMPK (AMP-activated protein kinase) and interacts with respiratory complex I, improving bioenergetics, reducing mitochondrial reactive oxygen species, and promoting mitophagy and organelle quality control. Importantly, despite distinct upstream mechanisms, NR and BBR converge on shared signaling pathways that support mitochondrial health, including redox balance, metabolic flexibility, and immunometabolic regulation. Unlike previous reviews addressing these compounds separately, this article integrates current preclinical and clinical findings to provide a unified perspective on their converging actions. We critically discuss translational opportunities as well as limitations, including heterogeneous clinical outcomes and the need for robust biomarkers of mitochondrial function. By outlining overlapping and complementary mechanisms, we highlight NR and BBR as rational combinatorial strategies to restore mitochondrial resilience. This integrative perspective may guide the design of next-generation clinical trials and advance precision approaches in mitochondrial medicine.

## 1. Introduction

### 1.1. General Context: The Central Role of Mitochondrial Function in Cellular Health

Mitochondrial dysfunction is increasingly recognized as a shared mechanistic denominator across ageing and multiple chronic disease areas. Beyond impaired ATP production, disrupted mitochondrial bioenergetics, redox control, and quality control are implicated in metabolic disorders (including type 2 diabetes, obesity and NAFLD/Non-Alcoholic Fatty Liver Disease), cardiovascular diseases (including atherosclerosis, ischemia–reperfusion injury and heart failure), neurodegenerative disorders (including Alzheimer’s and Parkinson’s disease), and ocular neurodegeneration, with glaucoma as a paradigmatic example. In this narrative review, these disease domains are discussed through a unifying lens: mitochondria act not only as metabolic organelles, but also as signaling hubs that integrate stress responses, inflammation, and tissue-specific vulnerability. Beyond their canonical role in ATP (adenosine triphosphate) generation, mitochondria are now recognized as central signaling hubs that couple metabolic status to innate immune effector functions. In phagocytes, mitochondrial pathways coordinate the switch between glycolysis and oxidative phosphorylation, the generation of mitochondrial reactive oxygen species (mtROS), changes in membrane potential and calcium fluxes, and the production of immunoregulatory metabolites such as succinate, fumarate and itaconate, thereby shaping macrophage and dendritic cell polarization, bactericidal activity, antigen presentation, inflammasome activation and efferocytosis [1,2,3,4,5,6].

A key layer of this crosstalk is provided by mitochondrial nucleic acids, particularly mitochondrial DNA (mtDNA), which behave as endogenous danger signals when they escape the matrix. Mechanistic studies show that mtDNA can be released through multiple, context-dependent routes—including BAK/BAX (BCL2 antagonist/killer 1/BCL2-associated X protein) “megapores”, mPTP–VDAC (mitochondrial permeability transition pore–voltage-dependent anion channel) oligomers, gasdermin-mediated damage and defective mitophagy or lysosomal clearance—and that different physical and chemical species of mtDNA (whole nucleoids versus fragments, oxidized versus non-oxidized, free or protein-bound) preferentially engage distinct pattern-recognition receptors such as cGAS–STING (cyclic GMP–AMP synthase–stimulator of interferon genes), NLRP3 (NLR family pyrin domain-containing 3), AIM2 (absent in melanoma 2) and TLR9 (Toll-like receptor 9) [7].

This mtDNA-driven signaling has been implicated in host defense against viral and bacterial infections, as well as in chronic inflammatory, autoimmune, cardiometabolic, and neurodegenerative diseases, and in ageing and senescence, highlighting “mitochondrial inflammatory signaling” as a shared mechanistic thread. At the same time, the precise code that links specific forms of mitochondrial stress to defined mtDNA species and immune pathways remains only partially understood, representing a major open question in the field [6,7].

### 1.2. Mitochondrial Dysfunction Across Metabolic and Neurodegenerative Disease, with Glaucoma as an Ocular Exemplar

Across metabolic and neurodegenerative disease contexts, mitochondria are increasingly recognized as signaling hubs that couple metabolic status to innate immune effector functions, including mtROS-dependent signaling and changes in membrane potential and calcium handling. In parallel, mitochondrial nucleic acids—particularly mtDNA—can act as endogenous danger signals when released through context-dependent routes and engage pattern-recognition receptors such as cGAS–STING, NLRP3 (NLR family pyrin domain containing 3), AIM2 and TLR9, linking mitochondrial stress to inflammatory signaling implicated in cardiometabolic and neurodegenerative disease, as well as ageing and senescence [1,2,3,4,5,6,7]. At the disease level, type 2 diabetes and related insulin-resistant metabolic disorders are associated with impaired mitochondrial oxidative metabolism, increased mtROS production, and alterations in mitochondrial dynamics/quality control, which contribute to insulin resistance and chronic low-grade inflammation [8,9,10]. In parallel, major neurodegenerative disorders (e.g., Alzheimer’s and Parkinson’s disease) are consistently associated with bioenergetic failure, defective mitochondrial quality control, and sustained neuroinflammatory signaling, placing mitochondrial homeostasis at the center of neurodegenerative pathophysiology [11,12]. Within this broader landscape, glaucoma provides an ocular exemplar in which mitochondrial vulnerability and stress-related signaling converge on retinal ganglion cells and their axons. The classical “pressure-only” model of glaucoma is increasingly being replaced by a neurodegenerative framework in which mitochondrial vulnerability is central. Work on hereditary mitochondrial optic neuropathies (Leber hereditary optic neuropathy or LHON, autosomal dominant optic atrophy or ADOA) has shown that primary defects of oxidative phosphorylation (OXPHOS) can selectively injure retinal ganglion cells (RGCs) despite ubiquitous expression of the mutated proteins [8,9,10,11,12]. Building on this, several reviews now argue that in glaucoma. Still, a mitochondrial dysfunction is not a late bystander, but a key factor in how RGCs respond to intraocular pressure (IOP), vascular dysregulation, and environmental stressors [13,14,15,16]. Clinically, many patients continue to lose vision despite adequate IOP control, suggesting that an intrinsically “fragile” mitochondrial phenotype can tip the balance towards neurodegeneration even when mechanical load is comparatively modest [13,14,15].

RGCs are among the most energy-demanding neurons of the CNS (central nervous system). Their large soma, extensive dendritic arbors, and long axons, with an unmyelinated intraocular segment, require continuous high-rate ATP production to sustain action potentials and axonal transport [13,14]. Mitochondria are densely clustered in these compartments, and ONH (optic nerve head) astrocytes harbor enlarged mitochondria to support axons crossing the lamina cribrosa, a site of high mechanical and metabolic stress [13,14,15]. RGC metabolism also depends on tight coupling with astrocytes through lactate shuttling and other substrate exchanges, so that mitochondrial dysfunction in either compartment can destabilize the whole retinal metabolic network [14,15,16]. This architectural and functional specialization explains why even relatively subtle bioenergetic defects can have disproportionate consequences on RGC survival.

Within this setting, three interconnected aspects of mitochondrial dysfunction recur across experimental and clinical studies: mtDNA (mitochondrial DNA) damage, oxidative stress, and defective quality control. Because mtDNA lies close to the respiratory chain and lacks the full nuclear DNA repair machinery, it is highly vulnerable to ROS (reactive oxygen species); increased oxidative DNA damage markers in aqueous humor and trabecular meshwork of POAG (primary open-angle glaucoma) patients indicate widespread mtDNA injury [13]. Ageing aggravates this process: in animal models, retinal NAD^+^ and glutathione decline with age, RGCs switch to less efficient metabolic pathways, and become more sensitive to IOP elevation. Blue-enriched light from LEDs (light-emitting diodes) and digital screens is increasingly discussed as a potential additional source of mitochondrial stress: experimental work suggests that short-wavelength light can be absorbed by flavoproteins and cytochromes, promoting ROS generation and impairing ATP synthesis in vulnerable retinal neurons. However, the magnitude and clinical relevance of this effect in human glaucoma remain to be fully defined [13,14]. At the genomic level, glaucoma cohorts show mtDNA copy number depletion and specific D-loop variants in subgroups of high-tension glaucoma, suggesting that altered regulation of mitochondrial replication may contribute to disease risk [16].

Mitophagy is the main selective quality-control mechanism that prevents dysfunctional mitochondria from accumulating. It relies on PINK1 (PTEN-induced kinase 1)–PARKIN–dependent ubiquitin signaling and on receptor-mediated pathways (e.g., BNIP3/NIX, AMBRA1) that tag damaged organelles for engulfment by autophagosomes and degradation in lysosomes. In glaucoma models, chronic IOP elevation leads to loss of mitochondria with normal membrane potential, cristae rarefaction, and reduced mitochondrial motility in ONH axons; impaired or insufficient mitophagy allows these depolarized, ROS-generating mitochondria to persist, amplifying oxidative stress [13,15].

Conversely, experimental overexpression of *PARKIN* or *OPA1* (optic atrophy 1) restores more physiological mitochondrial morphology, enhances PARKIN-mediated mitophagy, and protects RGCs against glaucomatous damage [13,14,15]. Importantly, some data suggest that both too little and too much mitophagy are harmful. In a chronic ocular hypertension model, fucoxanthin appears to dampen excessive mitophagy in the acute phase but to enhance PARKIN-mediated clearance during prolonged stress, supporting the idea that therapeutic benefit depends on re-establishing an appropriate mitophagy “set point” rather than simply increasing flux. Genetic studies reinforce the connection between mitophagy and glaucoma. Besides *MYOC* (myocilin), at least two Mendelian glaucoma genes, *TBK1* (TANK-binding kinase 1) and *OPTN* (optineurin), encode proteins that directly regulate PINK1–PARKIN signaling and LC3 (microtubule-associated protein 1A/1B-light chain 3) recruitment. Copy-number variants in *TBK1* have been detected in a subset of NTG (normal-tension glaucoma) across diverse ancestries, and OPTN mutations, such as E50K, are associated with more severe, early-onset NTG phenotypes [15].

A broader network of mitophagy- and dynamics-related genes shows association with POAG or NTG in at least some populations: *OPA1*, *MFN1* (mitofusin 1), *MFN2* (mitofusin 2), *DRP1* (dynamin-related protein 1), *AMBRA1* (Activating Molecule in Beclin-1-Regulated Autophagy 1), *CAV1/CAV2* (caveolin 1/caveolin 2), *HK2* (hexokinase 2), *UCP2* (uncoupling protein 2), *KEAP1/NRF2* (Kelch-like ECH-associated protein 1/nuclear factor erythroid 2–related factor 2), *TP53* (tumor protein p53) and *ROCK1* (Rho-associated coiled-coil containing protein kinase 1) all participate in mitochondrial fusion–fission cycles, mitophagy initiation or redox regulation [13,14,15,16]. However, many of these signals are modest and inconsistently replicated, and both positive and negative association studies exist for the same variants across different ethnic groups. Together with emerging data on mtDNA haplogroups in African-ancestry cohorts, these findings support a polygenic, ancestry- and environment-dependent contribution of mitochondrial pathways to glaucoma risk rather than a single “mitophagy gene” model [15,16].

These mechanistic insights are now informing mitochondria-targeted therapeutic strategies, although translation to clinical practice in glaucoma remains at an early stage. Antioxidants and mitochondrial-directed compounds such as vitamin B_3_ (nicotinamide), resveratrol, CoQ10, SkQ1, and SS-31 have shown promising neuroprotective effects in experimental glaucoma by improving mitochondrial function, reducing ROS, or modulating mitochondrial dynamics and mitophagy [13,14,15]. ROCK (Rho-associated coiled-coil containing protein kinase 1) inhibitors, already approved as IOP-lowering agents, can also enhance PARKIN recruitment and mitophagy via HK2 (hexokinase 2). At the same time, metformin appears to stimulate PINK1–PARKIN-dependent mitophagy and has been epidemiologically linked to reduced OAG incidence in diabetes. Gene therapy approaches using AAV2 (Adeno-Associated Virus serotype 2) vectors to deliver *OPA1* or *PARKIN* have protected RGCs in hypertensive models [13,15]. In parallel, clinical experience with idebenone, elamipretide, and ND4-replacement gene therapy in LHON provides proof-of-principle that modulating complex I function and mitochondrial biogenesis can alter visual outcomesin mitochondrial optic neuropathies [14,16].

At the same time, these approaches face substantial challenges. Much of the current evidence derives from in vitro or animal models that only partially reproduce human glaucoma, and negative or modestly positive trials in related conditions (e.g., memantine, idebenone) show that targeting mitochondria is not automatically sufficient to achieve clinically meaningful neuroprotection [13,14,15,16]. Both Yang et al. and Vallabh & Trounce stress the heterogeneity of RGC subtypes and disease phenotypes, warning against oversimplified classifications that obscure subtype-specific mechanisms and therapeutic windows. Future work will likely need to integrate mitochondrial genetics, functional read-outs, and advanced imaging, such as retinal flavoprotein fluorescence, to define mitochondrial endophenotypes within glaucoma and to guide genuinely personalized, mitochondria-based interventions [14,16].

### 1.3. Rationale of the Review: The Importance of Integrating NR and BBR; The Concept of Converging Mechanisms

Both nicotinamide riboside (NR) and berberine (BBR) have emerged from very different pharmacological traditions—one as a vitamin B_3_ -derived NAD^+^ precursor, the other as a quaternary isoquinoline alkaloid from traditional Chinese medicine—but the full-text evidence from our selected papers points to a surprisingly convergent target: mitochondrial quality control and its interface with inflammatory signaling. Across the NR studies included in this review, supplementation consistently increases intracellular NAD^+^ levels and activates NAD^+^-dependent deacetylases—especially sirtuins—thereby promoting PGC-1α signaling, mitochondrial biogenesis, and respiratory capacity in metabolically active tissues. In parallel, NR has been shown to reinforce adaptive stress responses, such as the mitochondrial unfolded protein response (UPRmt), and to facilitate mitophagy, thereby promoting the selective removal of damaged organelles and increasing mitochondrial resilience under metabolic, oxidative, or neurodegenerative stress [3,17,18,19]. These studies converge on a model in which NR primarily acts “upstream”, by restoring the NAD^+^ pool and re-energizing sirtuin/PGC-1α-dependent transcriptional programs that coordinate biogenesis, antioxidant defenses, and organelle turnover.

The BBR corpus we analyzed paints a complementary picture in which mitochondria are again central but targeted through distinct entry points. Fang et al. systematically map how BBR interacts with the mitochondrial respiratory chain, showing that it directly inhibits complex I in hepatocytes and myotubes, leading to reduced ATP synthesis, an increased AMP/ATP ratio, and subsequent activation of AMP-activated protein kinase (AMPK) [20]. The beneficial AMPK response discussed here is consistent with a partial and exposure-dependent attenuation of complex I flux; conversely, excessive or sustained inhibition would be expected to shift from adaptive signaling to bioenergetic compromise and cellular toxicity. In line with this, Fang et al. summarize that BBR toxicity is strongly influenced by systemic exposure and route of administration (e.g., much higher tolerated doses orally than intravenously) and appears to be linked to blood concentration [20], while clinical reviews report that typical oral doses used in metabolic indications are mainly associated with mild, transient gastrointestinal adverse events and show a dose–tolerability relationship in humans [21]. This AMPK-dependent signaling, together with more direct modulation of sirtuins (SIRT1 and SIRT3), drives PGC-1α activation, mitochondrial biogenesis, and reprogramming of substrate use, including enhanced fatty acid oxidation and reduced hepatic gluconeogenesis in models of obesity, diabetes, and non-alcoholic fatty liver disease [20,21]. Importantly, BBR does not simply “switch on” mitochondrial activity; rather, it remodels mitochondrial quality control. Experimental data summarized by Fang et al. show that BBR upregulates mitochondrial content and UCP1 (Uncoupling Protein 1)-mediated thermogenesis in brown and beige adipocytes, modulates fission/fusion dynamics via DRP1 (Dynamin-related protein 1), and promotes mitophagy through PINK1–Parkin and BNIP3/HIF-1α (BCL2 Interacting Protein 3/Hypoxia-Inducible Factor-1 alpha) pathways in cardiomyocytes, thereby limiting ischemia–reperfusion injury and pressure-overload–induced heart failure [20].

These mitochondria-centric actions extend beyond classical metabolic organs. Xu et al. review pharmacological and clinical data demonstrating that BBR improves insulin resistance, hyperlipidemia and NAFLD (Non-Alcoholic Fatty Liver Disease) by a combination of AMPK (AMP-activated protein kinase) activation, suppression of key gluconeogenic enzymes (PEPCK or Phosphoenolpyruvate carboxykinase, G6Pase or Glucose-6-phosphatase), enhancement of glycolysis, and stimulation of SIRT3 (Sirtuin 3)-dependent β-oxidation, while simultaneously correcting gut dysbiosis and intestinal barrier dysfunction [21]. Tian et al. further show that BBR reaches the central nervous system, crosses the blood–brain barrier and confers neuroprotection in models of Alzheimer’s disease, Parkinson’s disease, Huntington’s disease, ischemia–reperfusion and toxin-induced neurotoxicity. Mechanistically, BBR attenuates oxidative stress by directly scavenging multiple reactive species and by activating endogenous antioxidant pathways, including PI3K (Phosphatidylinositol 3-kinase)/Akt(also known as Protein kinase B) and PPARδ (Peroxisome Proliferator-Activated Receptor delta) driven Nrf2/HO-1 (Nuclear factor erythroid 2-related factor 2/Heme oxygenase-1) signaling, while restoring mitochondrial membrane potential and ATP levels and preventing cytochrome c and apoptosis-inducing factor release [22]. In neuronal systems, as in metabolic tissues, BBR thus acts as a modulator of mitochondrial function and redox homeostasis rather than a simple antioxidant.

A key added dimension emerging from the BBR literature is its impact on immunometabolism. Fang et al. highlight that BBR can induce mitophagy in macrophages, lowering mitochondrial ROS and thereby suppressing NOD-like receptor pyrin domain–containing protein 3 (NLRP3) inflammasome activation in the context of influenza infection [20]. Tian et al. summarize multiple in vitro and in vivo studies where BBR dampens neuroinflammation by inhibiting TLR4/MyD88/NF-Κb (Toll-Like Receptor 4/Myeloid Differentiation primary response 88/Nuclear Factor kappa-light-chain-enhancer of activated B cells) and MAPK (Mitogen-Activated Protein Kinase) signaling, downregulating iNOS (Inducible Nitric Oxide Synthase) and COX-2 (Cyclooxygenase-2), and reducing pro-inflammatory cytokines such as IL-1β (Interleukin-1β), IL-6 (Interleukin-6) and TNF-α (Tumor Necrosis Factor alpha), while increasing IL-4 (Interleukin-4) and IL-10 (Interleukin-10) [22]. In parallel, the mini-review by Chen et al. frames NLRP3 as a critical downstream effector of the cGAS–STING (cyclic GMP-AMP synthase- stimulator of interferon genes) cytosolic DNA-sensing pathway, which links mitochondrial damage and misplaced DNA to type I interferon responses, inflammasome activation, pyroptosis, and chronic inflammation across neurodegenerative, cardiovascular, hepatic, and renal diseases. STING can promote NLRP3 activation both by directly recruiting and deubiquitinating NLRP3 and by triggering lysosomal damage, cathepsin release, and K^+^ efflux [23]. Given this framework, the ability of BBR to limit mitochondrial ROS, promote mitophagy, and restrain NLRP3 activation positions it at a strategic node where mitochondrial quality control intersects with innate immune signaling.

Taken together, the NR and BBR literatures point to partially overlapping but mechanistically distinct routes towards the same therapeutic goal: restoring mitochondrial “fitness” and recalibrating the inflammatory tone in tissues vulnerable to metabolic and neurodegenerative injury. NR primarily augments the NAD^+^–sirtuin–PGC-1α axis, enhancing biogenesis, UPR^mt^ and mitophagy from a metabolic cofactor perspective [3,17,18,19], whereas BBR operates as a mitochondria-targeted xenobiotic that perturbs complex I, activates AMPK, modulates SIRT1/SIRT3 and PGC-1α, and shapes mitochondrial dynamics, mitophagy and redox signaling across liver, adipose tissue, kidney, heart and brain [20,21,22]. Both agents converge on core processes—bioenergetics, mitochondrial quality control, and immunometabolism—but the available studies have been conducted almost exclusively in isolation, with NR-focused papers rarely considering plant alkaloids, and BBR reviews largely ignoring NAD^+^ precursors and UPR^mt^ -centered interventions [3,17,18,19,20,21,22,23]. No work to date has systematically integrated these two strands to examine how NAD^+^ replenishment and mitochondria-targeted modulation might interact within shared pathways, including sirtuin/PGC-1α signaling, AMPK activation, mitophagy, NRF2-dependent antioxidant defenses, and the cGAS–STING–NLRP3 axis. This review is therefore grounded in the hypothesis that NR and BBR, by acting at complementary levels of the mitochondrial and innate immune networks described in the cited studies, could offer additive or synergistic benefits in conditions characterized by chronic mitochondrial stress and low-grade inflammation, and that mapping these convergences is a necessary step before rationally designing combined interventions.

It is important to emphasize that, to date, virtually no preclinical or clinical studies have systematically evaluated the combined administration of NR and BBR with prespecified mitochondrial or clinical endpoints. The present review, therefore, does not aim to summarize evidence on an established combination therapy but rather to develop a mechanistic framework that may inform the rational design of future NR–BBR cotreatment studies. Within this framework, any suggestion of additive or synergistic effects should be regarded as a hypothesis-generating interpretation of converging pathways, not as a conclusion drawn from direct combination trials.

### 1.4. Objectives and Scope of the Article

Taken together, recent comprehensive reviews underscore mitochondria and redox homeostasis as a shared therapeutic nexus across cardiovascular and neurodegenerative disorders. Work on brain aging and neurodegeneration has highlighted how age-related declines in NAD^+^ and impaired mitochondrial quality control contribute to synaptic failure, protein aggregation, and neuronal loss, and how pharmacological boosting of NAD^+^ with precursors such as nicotinamide riboside (NR), nicotinamide mononucleotide (NMN), or related compounds may restore mitochondrial resilience and attenuate neurodegenerative cascades [24].

In parallel, reviews in the cardiovascular field emphasize that NAD^+^/sirtuin signaling is deeply intertwined with mitochondrial metabolism, oxidative stress, and inflammation in atherosclerosis, ischemia–reperfusion injury, and heart failure, and propose NAD^+^ precursors as promising tools to improve cardiac bioenergetics and redox balance [25]. Complementary evidence from berberine research shows that this long-used botanical alkaloid exerts multi-level cardioprotective effects—ranging from AMPK activation and modulation of ROS-generating systems (e.g., NOX or Nitrogen Oxides, mitochondrial dysfunction) to anti-inflammatory and lipid-lowering actions—in preclinical models of coronary atherosclerosis, myocardial ischemia/reperfusion, arrhythmia and heart failure, with early clinical data supporting improvements in dyslipidemia and post-infarct inflammation. More broadly, a recent synthesis of mitochondrial-based therapies for neurodegenerative diseases details how strategies such as mitochondria-targeted antioxidants (e.g., MitoQ, SkQ1), PGC-1α-driven mitochondrial biogenesis, pharmacological enhancement of mitophagy, and even mitochondrial transplantation and gene-editing approaches are being explored to counteract mitochondrial dysfunction and oxidative stress in Alzheimer’s disease, Parkinson’s disease and amyotrophic lateral sclerosis; notably, this literature also classifies berberine among “exercise mimetics” that activate AMPK–PGC-1α signaling to improve mitochondrial function [12].

Building on these converging lines of evidence, the present work aims to integrate NAD^+^-boosting strategies and berberine-based interventions within a unified framework of mitochondrial and redox modulation, and to explore their potential complementarity across cardio- and neuro-centric conditions, with particular attention to disease settings where vascular, neurodegenerative, and immune-metabolic mechanisms intersect.

In addition to these cardio- and neuro-centric conditions, particular attention is given to ocular neurodegeneration, and glaucoma in particular, as a disease area where mitochondrial vulnerability, vascular dysregulation, and immunometabolic stress converge on retinal ganglion cells. By integrating data from systemic and ocular models, we aim to outline how NR- and BBR-centered strategies might eventually be positioned within mitochondria-targeted neuroprotection for chronic optic neuropathies.

### 1.5. Literature Search and Narrative Synthesis Strategy

We performed a targeted search of major bibliographic sources using combinations of keywords related to nicotinamide riboside and NAD^+^ metabolism (e.g., “nicotinamide riboside”, “NAD^+^, “sirtuin”, “PGC-1α”, “mitochondrial biogenesis”, “UPR^mt^ ”, “mitophagy”) and berberine (e.g., “berberine”, “complex I”, “AMPK”, “mitophagy”, “oxidative stress”, “Nrf2”, “mitochondrial dynamics”). Reference lists from key reviews and highly relevant primary papers were also screened to capture additional foundational and recent studies.

Studies were prioritized when they (i) provided mechanistic insight into mitochondrial pathways relevant to NR or BBR, (ii) reported mitochondrial-related functional outcomes in cellular or animal models, or (iii) presented clinical or translational data plausibly linked to mitochondrial endpoints (e.g., bioenergetics, redox markers, mitophagy/quality control readouts). Evidence was then charted and organized into thematic domains that guided the structure of the review: NAD^+^/sirtuin signaling and mitochondrial biogenesis; AMPK-centered stress adaptation; mitochondrial quality control (mitophagy, UPR^mt^, and dynamics); and redox/immunometabolic interfaces. This thematic mapping allowed an integrated comparison of NR and BBR across disease contexts and supported the converging mechanistic framework developed in Section 5.

## 2. Biological Background: Mitochondria, NAD^+^, and Quality Control

**Figure 1:**
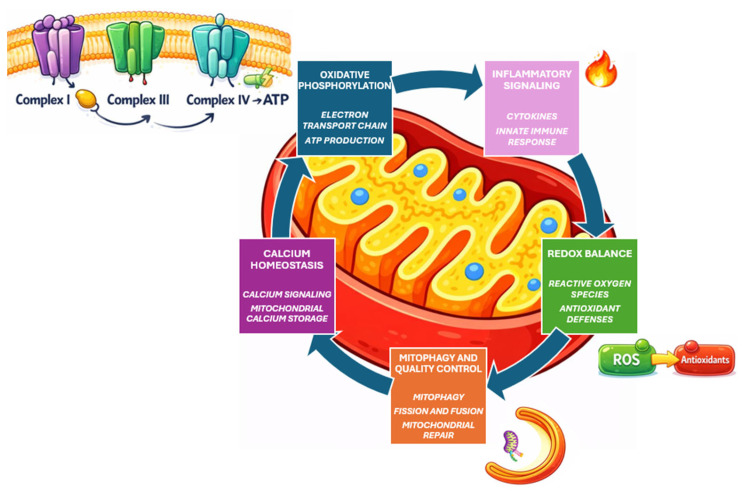
Core mitochondrial functions and stress-responsive pathways. Boxes indicate key functional domains (oxidative phosphorylation/ATP production, ROS–redox balance, Ca^2+^ homeostasis, mitochondrial quality control, and inflammatory signaling). Curved arrows depict their functional interdependence and feedback loops, whereby perturbation in one domain can propagate to others. Abbreviations: ATP, adenosine triphosphate; ROS, reactive oxygen species; Ca^2+^, calcium.

Figure 1 summarizes the core mitochondrial functions and stress-responsive pathways discussed in this section, including oxidative phosphorylation and ROS/redox balance, Ca^2+^ handling, mitochondrial quality control/mitophagy, and inflammatory signaling.

### 2.1. Bioenergetics: Respiratory Chain and Energy Production

Mitochondrial oxidative phosphorylation is organized around the mitochondrial respiratory chain (MRC), which consists of four main enzyme complexes (CI–CIV) embedded in the inner mitochondrial membrane, together with two mobile electron carriers, ubiquinone (CoQ) and cytochrome c (Cyt c). Electrons from metabolic substrates enter the chain primarily through CI (NADH dehydrogenase) or CII (succinate dehydrogenase), are passed to CoQ, then to the dimeric CIII, and finally to CIV via Cyt c. At CIV, molecular oxygen is reduced to water, and the resulting proton motive force is used by ATP synthase to drive ATP production. In this way, the respiratory chain provides both the structural scaffold and the functional core for mitochondrial ATP synthesis and for maintaining cellular metabolic homeostasis. However, electron transfer along the chain is not perfectly efficient. Electron leak at CI and CIII, particularly when membrane potential is high, or substrates are in excess, leads to the generation of mitochondrial reactive oxygen species (ROS). These ROS contribute to oxidative damage in cardiomyocytes and vascular cells and are now regarded as key mediators in a broad spectrum of cardiovascular diseases. Within this framework, the plant-derived isoquinoline alkaloid berberine has attracted attention as a potential cardioprotective agent: it reduces oxidative stress, improves endothelial function, and modulates redox-sensitive signaling pathways, further highlighting the close connection between respiratory chain activity, ROS production, and cardiovascular pathology [26,27,28,29].

The spatial organization of the respiratory chain complexes has been a matter of debate for many years. According to the traditional “solid” model, the complexes and carriers form a relatively rigid assembly in the inner membrane, maintaining tight physical contacts and high catalytic efficiency. In contrast, the “liquid” model describes the complexes as independent units that diffuse freely within the membrane, interacting functionally only through stochastic collisions of CoQ and Cyt c. In this scenario, electron transfer is viewed as a multi-collisional and comparatively slow diffusion-driven process. The identification of mitochondrial respiratory chain super complexes (SCs) by blue native PAGE in 2000 offered a way to reconcile these two views. SCs are higher-order assemblies composed of defined combinations of individual complexes—such as CI + CIII_2_, CI + CIII_2_ + CIV (the so-called “respirasome”), and CIII_2_ + CIV—which coexist with free complexes in the inner membrane. Structural and functional studies have shown that SCs are not artefacts of detergent solubilization, but genuine quaternary structures found across species and tissues. They appear to enhance electron transfer by shortening the effective diffusion distance for CoQ and Cyt c, to stabilize structurally fragile complexes, particularly CI, and to reduce electron leak and ROS formation [27,29].

Cardiomyocytes illustrate particularly well the tight coupling between respiratory chain architecture, energy demand, and redox balance. In the adult heart, mitochondria occupy a large proportion of the cell volume and must continuously adjust ATP production to the rapidly changing workload of each cardiac cycle. Kinetic models of oxidative phosphorylation in cardiomyocyte mitochondria explicitly incorporate the reactions of the respiratory complexes, ATP synthase, the proton motive force, substrate and ion transport, and feedback from cytosolic workload. These models show that relatively modest changes in complex activities, oxygen tension, or substrate supply can profoundly influence ATP content, membrane potential, and ROS output [28,29].

Together, experimental data and in silico analyses have led to the formulation of the “plasticity model. In this view, free complexes and SCs both contribute to electron transfer, and their relative abundance is dynamically tuned according to metabolic requirements and environmental conditions. The MRC is therefore understood not as a rigid assembly, but as a flexible network whose supramolecular organization can be remodeled to optimize energy conversion and redox control. Disruption of SC assembly has been associated with neurodegenerative diseases, cardiomyopathies, and inherited mitochondrial disorders, underscoring the essential role of proper SC organization in mitochondrial function and overall cellular homeostasis [29].

### 2.2. NAD^+^ as a Metabolic and Signaling Hub (Redox, Cofactor, Epigenetics)

Nicotinamide adenine dinucleotide (NAD^+^) is a central redox cofactor and, at the same time, an obligatory substrate for several enzyme families, including sirtuins, PARPs (Poly ADP-Ribose Polymerase), CD38/CD157, and SARM1 (Sterile Alpha and Toll/IL-1 Receptor Motif Containing 1). Through these interactions, NAD^+^ links cellular energy metabolism to DNA repair, chromatin remodeling, cellular senescence, circadian regulation, and immune responses [30,31,32].

NAD^+^ levels are maintained by a balance between synthesis and consumption, supported by partially overlapping biosynthetic routes. De novo synthesis from dietary tryptophan via the kynurenine pathway and the Preiss–Handler pathway from nicotinic acid runs in parallel with salvage pathways that recycle nicotinamide, nicotinamide riboside, and nicotinamide mononucleotide. Within these salvage routes, NAMPT (Nicotinamide Phosphoribosyl transferase) and the different NMNAT isoforms act as key rate-limiting enzymes [31,32]. This metabolism is not homogeneous across the cell: cytosolic, nuclear, and mitochondrial NAD+ pools are only partially interconnected, supporting local redox reactions and NAD^+^-dependent signaling.

A progressive decline in tissue NAD^+^ content is a consistent feature of ageing in both model organisms and humans. This reduction reflects a combination of reduced biosynthetic capacity and increased consumption, driven in particular by overactivation of PARPs and CD38 in the setting of chronic DNA damage, oxidative stress, and low-grade inflammation [30,31,32]. Human genetic studies make it clear that NAD^+^ is not just a passive marker but a causal hub: primary defects in enzymes of the de novo or salvage pathways, and secondary deficiencies due to impaired tryptophan transport, glutamine shortage, excessive NAD^+^ consumption or dietary niacin deficiency, underlie a wide clinical spectrum that includes congenital malformations, retinal degeneration, mitochondrial myopathy and neurodegeneration [32,33].

Across these conditions, NAD^+^ depletion is consistently linked to defective mitochondrial bioenergetics, impaired DNA damage responses, altered epigenetic regulation, and disturbed immunometabolic and cardiometabolic homeostasis. This convergence provides a strong rationale for therapeutic strategies aimed at restoring NAD^+^ availability. Nutritional interventions (vitamin B_3_ precursors), pharmacological approaches (NAD^+^ precursors, NAMPT activators, PARP or CD38 inhibitors), and lifestyle measures (caloric restriction, exercise) can all increase NAD^+^ levels and have shown beneficial effects in multiple preclinical models and early clinical trials. At the same time, important questions remain open regarding tissue specificity, long-term safety, and the potential pro-tumorigenic consequences of sustained NAD^+^ boosting [30,31,32,33].

### 2.3. Mitochondrial Quality Control Mechanisms

#### 2.3.1. Mitophagy (PINK1/Parkin and Alternative Pathways)

Mitophagy is an evolutionarily conserved, selective form of autophagy that eliminates dysfunctional or superfluous mitochondria, thereby maintaining mitochondrial quality and adjusting organelle mass to cellular metabolic demands. It relies on two partially overlapping modules: the ubiquitin-dependent PINK1/Parkin axis and a set of mitophagy receptors embedded in the outer or inner mitochondrial membrane. Under basal conditions, *PINK1* imported into healthy mitochondria is rapidly cleaved and degraded, whereas loss of membrane potential or accumulation of misfolded proteins stabilizes full-length *PINK1* on the outer membrane, where it phosphorylates ubiquitin and Parkin, triggering a feed-forward cascade of substrate ubiquitylation and recruitment of autophagy adaptors such as OPTN, NDP52 (Nuclear Dot Protein 52), p62 (also called Sequestosome-1 or SQSTM1), NBR1 (Neighbor of BRCA1 gene 1), and TAX1BP1 (Tax1-binding protein 1) [34,35,36].

In parallel, receptor-mediated mitophagy is driven by LC3 (Microtubule-associated protein 1 light chain 3)-interacting proteins including BNIP3 (BCL2 Interacting Protein 3), NIX/ BNIP3L, FUNDC1 (FUN14 Domain Containing 1), BCL2L13 (Bcl-2-like protein 13), FKBP8 (FK506 Binding Protein 8), PHB2 (Prohibitin 2) and cardiolipin, whose activity is tightly regulated by phosphorylation, ubiquitylation and localization, and often couples mitochondrial dynamics to mitophagosome formation. Fission–fusion proteins such as DRP1 (dynamin-related protein-1), MFN1/2 (Mitofusins 1/2), and OPA1, together with ER (Endoplasmic Reticulum)–mitochondria contact sites, shape mitochondrial networks and prime damaged segments for selective engulfment. However, fragmentation per se is not always sufficient to induce mitophagy. Physiologically, mitophagy supports basal mitochondrial turnover, prevents clonal expansion of mutant mtDNA, and is essential in developmental contexts such as erythroid maturation, retinal ganglion cell differentiation, muscle regeneration, and paternal mitochondria clearance [34,35].

Conversely, defective or insufficient mitophagy contributes to neurodegeneration, particularly in PINK1- or Parkin-linked Parkinson’s disease, and to exaggerated inflammatory signaling via mtROS and mtDNA-driven inflammasome and cGAS–STING activation [36].

In the kidney, where energetic demand is high, dysregulated mitophagy is increasingly recognized as a central mechanism in acute kidney injury, diabetic kidney disease, and lupus nephritis, making pharmacological modulation of mitophagy an attractive therapeutic strategy [36,37].

#### 2.3.2. Mitochondrial Unfolded Protein Response (UPR^mt^)

The mitochondrial unfolded protein response (UPR^mt^) is an adaptive transcriptional program that preserves organelle function when mitochondrial proteostasis and import are perturbed. In *Caenorhabditis elegans*, accumulation of unfolded proteins and impaired oxidative phosphorylation reduce mitochondrial import efficiency, leading to export of peptide fragments through HAF-1 (Hypoxia-Associated Factor-1) and failure of the Bzip (basic Leucine Zipper) transcription factor ATFS-1 (Activating Transcription Factor associated with Stress 1) to enter the matrix. Instead, ATFS-1, which carries both a weak mitochondrial targeting sequence and a nuclear localization signal, accumulates in the nucleus where, together with DVE-1 (Developmental, Vital, and Energy-related 1) and UBL-5 (Ubiquitin-Like protein 5), it induces a broad gene-expression program encompassing mitochondrial chaperones and proteases, antioxidant enzymes, protein-import components, and metabolic regulators. Chromatin remodeling by MET-2 (methyltransferase 2)/LIN-65, JMJD-3.1 (Jumonji domain–containing histone demethylase-3.1), JMJD-1.2 (Jumonji domain–containing histone demethylase-1.2), and the co-activator CBP-1 (CREB-binding protein 1) generates a permissive epigenetic landscape that restricts this response to specific loci, functionally coupling mitochondrial stress sensing to nuclear transcription. A parallel reduction in global translation via eIF2α (eukaryotic translation initiation factor 2 alpha) phosphorylation optimizes the folding environment in stressed mitochondria and limits further influx of precursors. In mammals, UPR^mt^ signaling is organized around a canonical DELE1 (DAP3-binding cell death enhancer 1)–HRI (heme-regulated inhibitor)–integrated stress response axis. Under proteotoxic stress, the inner-membrane protease OMA1 (Mitochondrial Protease 1) cleaves DELE1, allowing short DELE1 fragments to accumulate in the cytosol and activate the eIF2α kinase HRI. Phosphorylated eIF2α selectively enhances translation of ATF4 (activating transcription factor 4), CHOP (C/EBP homologous protein), and ATF5 (activating transcription factor 5) while globally attenuating protein synthesis. ATF5, whose mitochondrial import is also compromised during stress, translocate to the nucleus and, together with ATF4, CHOP and HSF1 (heat shock factor 1), drives expression of nuclear-encoded mitochondrial chaperones (HSP60 or heat shock factor 60, HSP10 or heat shock factor 10, mtHSP70 or mitochondrial heat shock protein 70), proteases (LONP1 or Lon peptidase 1, CLPP or caseinolytic mitochondrial matrix peptidase proteolytic subunit, YME1L1 or *YME1-like 1 ATP-dependent metalloprotease*), antioxidant defenses and metabolic genes. Additional UPR^mt^ “axes”, including a SIRT3 (*sirtuin 3*)–FOXO3a (forkhead box O3a)-dependent antioxidant branch and an intermembrane-space UPR involving Erα (estrogen receptor alpha) and NRF1 (nuclear respiratory factor 1), further diversify this response. Collectively, these pathways maintain mitochondrial proteostasis, support metabolic rewiring, and contribute to organismal stress resistance and longevity, but chronic or dysregulated UPR^mt^ activation may also sustain cancer cell survival or aggravate tissue damage, underscoring its context-dependent role in disease [38,39,40].

#### 2.3.3. Mitochondrial Dynamics: Fusion, Fission, and Biogenesis

Mitochondria are plastic organelles that remodel through coordinated cycles of fusion, fission, and biogenesis to adapt number, morphology, and localization to cellular energetic and signaling needs. In mammalian cells, outer-membrane fusion is mediated by the dynamin-related GTPases mitofusin-1 and -2 (MFN1, MFN2), inner-membrane fusion by optic atrophy 1 (OPA1). In contrast, fission is driven by dynamin-related protein-1 (DRP1), recruited to the outer membrane by adaptors such as FIS1 (Mitochondrial fission 1 protein), mitochondrial fission factor (MFF), and MiD49/51 (Mitochondrial dynamics protein of 49/51 kDa). These core effectors are tightly regulated by post-translational modifications, linking mitochondrial morphology to metabolic stress, Ca^2+^ signaling, and the cell cycle [2,41,42].

Beyond reshaping the network, fusion–fission cycles are central to mitochondrial quality control. Individual fission events can be functionally asymmetric, generating daughter organelles with divergent membrane potentials; more depolarized fragments are excluded from subsequent fusion and preferentially targeted to PTEN-induced kinase-1 (PINK1)–Parkin–mediated mitophagy, whereas better-preserved daughters reintegrate into the functional pool. In cardiomyocytes, the fusion protein MFN2 occupies a nodal position in this interactome. Upon PINK1-dependent phosphorylation, it can act as a Parkin receptor on damaged mitochondria, directly coupling outer-membrane fusion machinery to selective mitophagy [42].

Mitochondrial biogenesis provides the complementary anabolic arm of this system. In cardiomyocytes, the transcriptional coactivators PGC-1α and PGC-1β coordinate a nuclear program that activates nuclear respiratory factors (NRF-1, NRF-2), estrogen-related receptors (ERRs), and mitochondrial transcription factors, such as TFAM (Mitochondrial Transcription Factor A), thereby expanding mitochondrial DNA copy number, respiratory-chain subunit levels, and oxidative capacity. During perinatal and postnatal cardiac maturation, this PGC-1-driven program accompanies the metabolic shift from glycolysis to fatty-acid β-oxidation and a marked increase in mitochondrial content and size. [41] Genetic disruption of PGC-1 coactivators or of key fusion–fission proteins (MFN1/2, OPA1, DRP1) results in defective mitochondrial biogenesis, impaired cardiomyocyte maturation, and cardiomyopathy, indicating that balanced fusion, fission, and biogenesis are prerequisites for normal cardiac development and function [41,42].

#### 2.3.4. Mitochondrial-Driven Cell Death (mPTP, Apoptosis, mtDNA Release)

Mitochondria are increasingly framed as the central decision-makers between adaptation and demise, with the mitochondrial permeability transition pore (mPTP) and mitochondria-associated programmed cell death (PCD) pathways representing two tightly interconnected layers of this control system. Bernardi et al. describe mPT (mitochondrial permeability transition) as a Ca^2+^ and cyclophilin D dependent increase in inner mitochondrial membrane permeability mediated by a high-conductance, non-selective channel whose persistent opening drives mitochondrial swelling, outer membrane rupture, release of cytochrome c and other pro-death factors, and ultimately apoptotic or necrotic cell death, while brief, low-conductance openings contribute to physiological regulation of bioenergetics, Ca^2+^ homeostasis and ROS signaling. Their analysis underscores that the exact molecular identity of the mPTP (mitochondrial permeability transition pore) remains unresolved, with converging but still debated evidence implicating F_1_F_0_-ATP synthase (dimers, monomers or c-ring) and the adenine nucleotide translocase as pore-forming entities modulated by CypD (cyclophilin D), and highlights major technical obstacles (protein fragility, low fraction of pore-competent complexes, limitations of current cryo-EM (cryo-electron microscopy) and in vitro systems) that complicate structure–function studies and drug development [43].

In parallel, Nguyen et al. broaden this perspective by situating mitochondria within an integrated quality-control network that includes mitohormesis, the mitochondrial unfolded protein response (UPR^mt^), mitokines (e.g., GDF15 or growth differentiation factor 15, FGF21 or *fibroblast growth factor 21*, MOTS-c or *mitochondrial open reading frame of the 12S rRNA*, humanin) and mitophagy, and by systematically reviewing how mitochondrial dysfunction interfaces with multiple forms of PCD or programmed cell death—apoptosis, necroptosis, ferroptosis, pyroptosis, parthanatos and paraptosis—each defined by distinct triggers and executioner machineries but converging on mitochondrial stress, membrane permeabilization and loss of bioenergetic competence in age-related neurodegenerative, cardiovascular, metabolic and neoplastic diseases. Their work also maps a growing repertoire of druggable nodes along these pathways (BCL-2 or B-cell lymphoma *2* family proteins, RIPK1 (receptor-interacting serine/threonine-protein kinase 1)/RIPK3 (*receptor-interacting serine/threonine-protein kinase 3*)–MLKL (mixed lineage kinase domain-like pseudokinase), GPX4 (glutathione peroxidase 4)/system xC^−^ (cystine/glutamate antiporter), NLRP3–gasdermin (*NLR family pyrin domain containing 3*), PARP1) and summarizes early-phase clinical trials that target mitochondrial quality control or death signaling in humans [44].

Taken together, these contributions and other recent evidence [45,46] support a unifying model in which Ca^2+^ overload, ROS production, defective MQC and context-dependent mPTP opening do not act as isolated events, but as components of a dynamic, interconnected network that sets the threshold between adaptive mitochondrial stress responses and irreversible PCD during aging and disease; therapeutically, this suggests that effective interventions will likely need to combine stabilization of mPTP-forming complexes with enhancement of mitochondrial quality control and selective modulation of downstream PCD pathways rather than focusing on a single effector in isolation [43,44,45,46].

### 2.4. Mitochondria and Immunometabolism (cGAS–STING, NLRP3 Inflammasome)

Because of their bacterial ancestry, mitochondria sit at a strategic crossroads between metabolism and innate immunity. They host oxidative phosphorylation, TCA (tricarboxylic acid) cycling, and biosynthetic pathways, but also contain damage-associated molecular patterns (DAMPs) such as mtDNA, cardiolipin, N-formyl peptides, and ROS that can be released upon stress or mitochondrial outer membrane permeabilization (MOMP). Once in the cytosol or extracellular space, mtDNA and other mitochondrial DAMPs engage pattern-recognition receptors, notably the DNA sensor cGAS (cyclic GMP–AMP synthase) and the NLRP3 (NLR family pyrin domain containing 3) inflammasome, converting metabolic damage into type I interferon and IL-1 (Interleukin-1)–family cytokine production [7,47,48].

Mechanistically, mtDNA can escape the matrix through several inducible pores. BAX/BAK “megapores” formed during apoptosis or minority MOMP allow herniation of the inner membrane and extrusion of whole nucleoids, whereas mPTP–VDAC (voltage-dependent anion channel) macropores preferentially release oxidized mtDNA fragments; defective mitophagy and lysosomal dysfunction further favor leakage of mitochondrial material [7,47].

Cytosolic mtDNA activates cGAS, which generates cGAMP and triggers STING (stimulator of interferon genes)–TBK1 (*TANK-binding kinase 1*)–IRF3 (*interferon regulatory factor 3*) signaling, leading to interferon-stimulated gene expression and NF-κB activation; this axis can both prime and trigger NLRP3, providing “signal 1” (transcriptional upregulation of NLRP3 and pro-IL-1β) and, in some contexts, “signal 2” via lysosomal and ionic stress. Inflammatory macrophages exemplify the immunometabolic coupling of these pathways: TLR ligation drives a shift from OXPHOS (oxidative phosphorylation) to glycolysis, mitochondrial depolarization, and mitoROS; enhanced mtDNA synthesis (via CMPK2 ort cytidine/uridine monophosphate kinase 2) generates poorly protected templates that accumulate oxidative lesions, are processed into fragments, and exported through mPTP/VDAC to activate NLRP3 and cGAS–STING in parallel [7,48].

Persistent mtDNA release and mitochondrial dysfunction therefore integrate metabolic rewiring with innate immune signaling, contributing to chronic inflammatory, cardiovascular, and neurodegenerative diseases while also amplifying host defense against infection [7,47,48].

Although the focus above is on innate immune sensing (cGAS–STING and the NLRP3 inflammasome), mitochondrial metabolism and NAD^+^ availability are also central to adaptive immunity. T-cell activation, differentiation, and long-term function require rapid metabolic rewiring, and several NAD+-consuming enzymes (including sirtuins, PARPs, and CD38) have been implicated in shaping T-cell fate decisions and inflammatory output. Accordingly, NAD^+^-boosting strategies, such as nicotinamide riboside, may also influence T-cell biology. However, a full discussion of adaptive immunity is beyond the scope of this review [30,31,32].

## 3. Nicotinamide Riboside (NR): Mechanisms, Preclinical and Clinical Evidence

### 3.1. Chemistry, Pharmacokinetics, and Bioavailability

Nicotinamide riboside (NR) is a pyridine-nucleoside form of vitamin B_3_ and a salvageable NAD^+^ precursor. It is present in foods such as milk and arises in the gut from NAD^+^, which is hydrolyzed to NMN, then to NR before uptake. Intracellularly, NR is converted to NMN by NRK1/NRK2 (Nicotinamide riboside kinase 1/2) and then to NAD+ by NMNAT (Nicotinamide mononucleotide adenylyl transferase) isoforms, providing a two-step (or, via purine nucleoside phosphorylase plus NAMPT, three-step) pathway that bypasses the rate-limiting NAMPT step and the need for PRPP (5-Phosphoribosyl-1-pyrophosphate). This route is important in NAMPT-expressing cells, where NAMPT expression declines under metabolic stress, and in nervous tissues, where *NRK2* is induced by axonal injury and heart failure [49,50,51].

Crystalline NR chloride (NIAGEN^®^) is orally bioavailable and well-tolerated in humans, as shown in a randomized, double-blind, placebo-controlled trial in healthy, overweight adults [50]. NIAGEN^®^ has also undergone preclinical safety evaluation, including acute, 14-day, and 90-day rat toxicology studies (NOAEL 300 mg/kg/day; LOAEL 1000 mg/kg/day), as reported in the Introduction of the same study [50,51].

In humans, single and repeated oral doses (100–1000 mg/day) produce dose-dependent rises in whole-blood NAD^+^ (≈10–140% over baseline) and in NAM (Nicotinamide), MeNAM (N^1^-methylnicotinamide (also written 1-methylnicotinamide) and Me2PY (N^1^-methyl-2-pyridone-5-carboxamide), without flushing or changes in LDL-cholesterol or homocysteine [50]. Trials using up to 2 g/day for 12 weeks reported no serious adverse events [50,51].

NR bioavailability is tissue-dependent and shaped by NRK expression: liver, skeletal muscle, brown fat, and heart show robust NAD*^+^* responses, whereas brain and white adipose tissue respond less consistently.

Although NR is unstable in plasma and is partly degraded to NAM in the gut and liver, repeated oral dosing reliably elevates the NAD^+^ metabolome, including NAAD (Nicotinic acid adenine dinucleotide), supporting NR as an effective NAD^+^-boosting agent in vivo [50,51].

NAD^+^ salvage pathway: NR → NMN → NAD^+^.

In mammals, most tissues maintain NAD^+^ mainly through salvage pathways, whereas de novo synthesis from tryptophan is largely confined to the liver and the kidney. Nicotinamide riboside (NR) is a vitamin B_3_ precursor that enters cells via equilibrative nucleoside transporters, feeding the salvage network. Inside the cell, NR is phosphorylated by nicotinamide riboside kinases NRK1 and NRK2 to form nicotinamide mononucleotide (NMN), which is then adenylated by NMNAT1–3 to generate NAD^+^, defining the NR → NMN → NAD^+^ route [3,49,50,51,52].

NR can also be cleaved by purine nucleoside phosphorylase to nicotinamide (NAM); NAM is converted to NMN by NAMPT and then to NAD^+^ by NMNAT isoenzymes, functionally connecting NRK- and NAMPT-dependent salvage. These reactions are compartmentalized: NMNAT1 is nuclear, NMNAT2 cytosolic/Golgi, and NMNAT3 mitochondrial, supporting distinct subcellular NAD^+^ pools [3].

NRK1 is widely expressed, whereas NRK2 shows a more restricted pattern, so the contribution of NR to NAD^+^ salvage is tissue-specific [3,52]. Beyond cell-autonomous pathways, NAMPT exists extracellularly (eNAMPT), producing NMN from circulating NAM; NMN can be dephosphorylated to NR, imported, and reconverted to NAD+, while NAD+ glycohydrolases, including CD38, generate NAM, NR, and NMN outside cells that are recycled by salvage. Tracer studies show that NR and NMN are partly converted to NAM, deamidated by the gut microbiota into nicotinic acid, and returned via enterohepatic circulation to the liver, where they enter the Preiss–Handler pathway [3].

Together, these findings indicate that the NR → NMN → NAD^+^ pathway is a nodal component of a broader salvage network integrating NRK, NAMPT, and Preiss–Handler routes with microbiome-dependent metabolism [49,50,51,52].

### 3.2. Molecular Mechanisms of Action

#### 3.2.1. Sirtuin Activation (SIRT1, SIRT3)

Sirtuins are NAD^+^-dependent deacetylases, with SIRT1 mainly nuclear and SIRT3 mitochondrial, and their activity is limited by NAD^+^ availability [17,53,54]. Nicotinamide riboside (NR) is a NAD^+^ precursor that increases intracellular and mitochondrial NAD^+^ in mammalian cells and raises NAD^+^ in mouse liver and skeletal muscle, thereby enabling sirtuin activation. In cultured myotubes and hepatocytes, NR dose-dependently lowers FOXO1 (Forkhead Box O1) acetylation in a SIRT1-dependent manner, without changing SIRT1 protein levels, suggesting activation of pre-existing SIRT1 by increased NAD+ and leading to the induction of FOXO1 target antioxidant genes and the repression of UCP2 (Uncoupling Protein 2). In vivo, NR-driven NAD^+^ elevation is associated with deacetylation of SIRT1 targets FOXO1 and PGC-1α, as well as the SIRT3 target SOD2 (Superoxide dismutase 2), with parallel induction of oxidative metabolism genes, mitochondrial DNA, and respiratory chain proteins, indicating enhanced mitochondrial biogenesis and oxidative capacity [17]. In ataxia-telangiectasia models, persistent DNA damage causes PARP1 hyperactivation, NAD^+^ depletion, and reduced SIRT1 activity, whereas NAD^+^ replenishment with NR or related interventions restores SIRT1-dependent deacetylation, improves mitochondrial function and mitophagy, and prolongs health span [53]. NR also elevates mitochondrial NAD^+^ and deacetylates the SIRT3 substrates NDUFA9 (NADH dehydrogenase (ubiquinone) 1 alpha subcomplex, 9) and SOD2, increasing SOD2 activity, while SIRT3-deficient cells fail to deacetylate SOD2 despite similar NAD^+^ increases, indicating direct SIRT3 activation by NR [17,18,19,20,21,22,23,24,25,26,27,28,29,30,31,32,33,34,35,36,37,38,39,40,41,42,43,44,45,46,47,48,49,50,51,52,53]. SIRT3 deacetylates FOXO3 and SOD2 to strengthen antioxidant defenses and induce mitophagy-related genes (NIX or NIP3-like protein X, BNIP3 or BCL2/adenovirus E1B 19 kDa-interacting protein 3, LC3 or Microtubule-associated protein 1A/1B-light chain 3), and age-related declines in NAD^+^ and sirtuin activity can be counteracted experimentally by NAD^+^ precursors such as NR, which improve mitochondrial quality via mitophagy in models of aging and neurodegeneration [17,53,54].

#### 3.2.2. PGC-1α and Mitochondrial Biogenesis

PGC-1α is a key regulator of mitochondrial biogenesis whose activity is controlled by NAD^+^-dependent deacetylation by SIRT1. In an ethanol-induced alcoholic liver disease model, chronic alcohol exposure lowers hepatic NAD^+^ and SIRT1, increases PGC-1α acetylation, reduces its nuclear localization, and is accompanied by loss of mitochondrial content, impaired TCA cycle enzymes, decreased oxygen consumption and ATP, and upregulation of UCP2 (Uncoupling protein 2). Nicotinamide riboside (NR), by replenishing NAD^+^, restores SIRT1 expression and deacetylase activity, promotes PGC-1α deacetylation and nuclear translocation without changing total PGC-1α, and upregulates the downstream ERRα (Estrogen-related receptor alpha)–NRF1/NRF2 (Nuclear respiratory factor 1/2)–TFAM program, thereby increasing mtDNA copy number, oxidative phosphorylation, and energy production. Genetic or pharmacological inhibition of SIRT1 abolishes NR-induced mitochondrial improvements and aggravates steatosis, and human alcoholic livers show reduced SIRT1 activity, enhanced PGC-1α acetylation, and lower NRF1/TFAM expression, mirroring these findings. In *POLG* (DNA polymerase subunit gamma)—mutant astrocytes, mitochondrial mass, membrane potential and the biogenesis coactivator PGC-1β are reduced; combined NR and metformin treatment activates SIRT1 and AMPK, attenuates Mtor (Mammalian Target of Rapamycin) signaling, increases PGC-1α expression, and improves mitochondrial mass, membrane potential and complex I expression, while low-dose metformin also enhances complex I activity [55,56].

#### 3.2.3. UPR^mt^ Activation and Stress Responses

The mitochondrial unfolded protein response (UPR^mt^) is a conserved stress program triggered by the accumulation of unfolded proteins in the mitochondrial matrix. In mammals, canonical UPR^mt^ signaling involves OMA1-mediated cleavage of DELE1 (DAP3 Binding Cell Death Enhancer 1), activation of the HRI (Hematopoietic Receptor Interactor)–eIF2α (eukaryotic initiation factor 2α) integrated stress response, and increased translation of ATF5 (Activating Transcription Factor), ATF4 (Activating Transcription Factor 4) and CHOP (Cyclophosphamide, Hydroxydaunorubicin (Doxorubicin), Oncovin (Vincristine), and Prednisone), which induce mitochondrial chaperones (Hsp60, Hsp10, mtHsp70 or Mitochondrial Heat Shock Protein 70) and proteases (LONP1 or Lon Peptidase 1, Mitochondrial, ClpP or Caseinolytic Protease P) after chromatin remodeling by histone demethylases and the co-activator CBP/p300 (CREB-Binding Protein and p300). Additional axes include a local translational response that reduces mitochondrial protein synthesis, a SIRT3–FOXO3a pathway that upregulates antioxidant enzymes, and an intermembrane-space UPRIMS (Unfolded Protein Response of the InterMembrane Space)/ERα branch driven by ROS-dependent AKT/PKB (Protein kinase B) activation. UPR^mt^ activation has been reported in conditions with mitochondrial dysfunction, including neurodegenerative diseases, where it can preserve ATP production and limit ROS, although chronic activation may become detrimental. In *SOD1G93A* ALS mice, mitochondrial proteostasis is disrupted, and UPR^mt^ markers (LONP1 or Lon peptidase 1, HSP60, CLPP or Caseinolytic Peptidase P) are upregulated in brain tissue.

Oral NR supplementation further increases these UPR^mt^ -related proteins, promotes clearance of mitochondrial mutant hSOD1, and enhances NSC/NPC (Neural Stem Cells/Neural Progenitor Cells) proliferation and adult neurogenesis, without delaying disease onset or extending survival.

These data indicate that pharmacological boosting of NAD*^+^* can engage UPR^mt^-mediated stress responses to partially restore mitochondrial proteostasis and neural plasticity in neurodegenerative settings [40,57].

#### 3.2.4. Mitophagy Regulation and Organelle Turnover

Mitophagy is a crucial component of mitochondrial quality control, eliminating damaged organelles. In alcohol-induced neuronal injury, impaired mitophagy and disrupted UPR^mt^ act as key drivers of mitochondrial dysfunction, ROS accumulation, and cognitive decline. Nicotinamide riboside chloride (NRC) restores mitochondrial homeostasis by enhancing UPR^mt^ programs and Fundc1-dependent mitophagy, normalizing ATP production, complex IV activity, membrane potential, and mitochondrial morphology. Pharmacological inhibition of UPR^mt^ abolishes NRC-mediated Fundc1 upregulation and mitophagy, whereas blocking mitophagy does not prevent UPR^mt^ activation, indicating a hierarchical UPR^mt^ → Fundc1 axis controlling organelle turnover [58].

In ataxia telangiectasia models, *ATM* deficiency leads to defective mitophagy, accumulation of damaged mitochondria and cytoplasmic mtDNA, STING-driven senescence, and SASP (Senescence-Associated Secretory Phenotype). Raising NAD*^+^* with nicotinamide riboside stimulates PINK1-dependent mitophagy, clears dysfunctional mitochondria, reduces cytoplasmic DNA and inflammatory signaling, and improves neuronal survival and motor performance [59].

Collectively, these data support targeting mitophagy pathways to preserve mitochondrial integrity and neuronal function [58,59].

#### 3.2.5. Redox Balance, ROS Control, and Mitochondrial DNA Repair

In primary human fibroblasts, basal mitophagy is driven by mitochondrial superoxide, with PINK1/Parkin/p62 targeting superoxide-enriched mitochondria for autophagic removal. Senescence triggers an early shutdown of ROS-initiated mitophagy, leading to the accumulation of dysfunctional mitochondria, bioenergetic decline, and cellular aging phenotypes, whereas pharmacological mitophagy activators, including NAD^+^ precursors and a p62-oligomerizing small molecule, can partially reverse these changes [60].

In Werner syndrome, *WRN* mutations associate with mitochondrial ROS accumulation, ATP depletion and NAD^+^ loss, together with defective mitophagy and shortened lifespan in worm and fly models; NAD^+^ repletion with nicotinamide riboside or nicotinamide mononucleotide normalizes NAD^+^ metabolic profiles restoring depleted NAD^+^ levels via NR or NMN supplementation normalizes the NAD^+^ metabolome, restores DCT-1/ULK-1 (Dauer formation- and Cold acclimation-related gene 1/UNC-51-like kinase 1)–dependent mitophagy, improves mitochondrial morphology, extends lifespan, and enhances homologous recombination capacity after DNA damage [61].

Consistently, nicotinamide riboside supplementation in mammalian systems elevates mitochondrial NAD^+^, activates SIRT1 and SIRT3, increases SOD2 (Superoxide dismutase 2) activity, and globally enhances oxidative metabolism [17].

Overall, these findings indicate that fine-tuning mitochondrial ROS signaling and boosting NAD^+^ availability converge on mitochondrial quality control and redox homeostasis, with potential to alleviate both cellular senescence and systemic metabolic aging.

### 3.3. Preclinical Evidence

Preclinical studies of nicotinamide riboside (NR) span complementary cellular and animal models. In Tg2576 Alzheimer models, NR elevates cortical NAD*^+^*, improves cognitive performance, restores hippocampal long-term potentiation and, in primary cortical–hippocampal neurons, upregulates PGC-1α, enhances BACE1 (Beta-site amyloid precursor protein cleaving enzyme 1) ubiquitination and proteasomal degradation, and reduces Aβ1–40/42 (Amyloid-beta peptides 1–40 and 1–42) release; PGC-1α silencing or knockout abolishes these effects [62].

In non-neuronal systems, NR raises NAD^+^ in C2C12 myotubes, Hepa1.6 and HEK293T cells, increases mitochondrial NAD+ in HEK293T cells, and activates SIRT1/SIRT3-dependent deacetylation of FOXO1, NDUFA9 (NADH:ubiquinone oxidoreductase subunit A9), and SOD2, as confirmed in *SIRT3* ⁺/⁺ versus *SIRT3* ^−^/^−^ *MEFs* (Mouse embryonic fibroblasts). In vivo, dietary NR increases NAD+ levels in the liver and muscle, upregulates genes involved in energy metabolism, enhances energy expenditure, and mitigates high-fat diet–induced obesity and insulin resistance [17].

Systemic NR also elevates retinal NAD^+^, preserves pattern ERG responses and retinal ganglion cell survival after optic nerve crush, limits GFAP (Glial fibrillary acidic protein) -positive gliosis, and maintains RGC counts despite intraocular pressure elevation in microbead-induced ocular hypertension [63].

### 3.4. Clinical Studies and Human Trials

Most of the clinical work with nicotinamide riboside (NR) has focused on safety, pharmacodynamic effects on NAD^+^, and exploratory signals of efficacy rather than hard clinical endpoints. In a 6-week randomized, double-blind, placebo-controlled crossover trial in healthy middle-aged and older adults, 1000 mg/day of NR was well tolerated and raised NAD^+^ levels in peripheral blood mononuclear cells by roughly 60%. Beyond this clear biochemical effect, only modest trends were observed: systolic blood pressure and aortic stiffness tended to decrease in participants with higher baseline values, while there were no convincing changes in body composition, glucose homeostasis, exercise capacity, or motor performance [64].

A recent critical review of 25 human NR studies draws a similar picture. Across heterogeneous populations and study designs, oral NR consistently increases circulating NAD^+^-related metabolites, but reproducible, clinically meaningful effects remain limited. The most consistent signals emerge in the modulation of inflammatory markers and in a few severe disease settings, whereas broad cardiometabolic benefits have not been firmly established so far [65].

Parkinson’s disease is one of the areas where NR has been explored more intensively. In early-phase trials, 1000 mg/day of NR has been associated with increases in cerebral NAD^+^ and with neurometabolic and transcriptional changes compatible with target engagement. In the NR-SAFE phase I study, doses up to 3000 mg/day for 4 weeks were well tolerated and produced approximately 3–5-fold increases in blood NAD^+^ and NADP^+^. Treatment was accompanied by only a mild, non-progressive rise in homocysteine and by indications of symptomatic improvement. However, the latter may be partly confounded by concomitant levodopa timing and therefore must be interpreted with caution [65,66].

From an ophthalmic perspective, a multicenter, randomized, double-blind trial is currently underway to assess 300 mg/day of NR for 24 months in patients with primary open-angle glaucoma. The study uses retinal nerve fiber layer thinning and visual field outcomes to evaluate whether NR can provide structural neuroprotection and short-term neuroenhancement in patients with chronic optic neuropathy [67].

### 3.5. Limitations and Controversies (Heterogeneous Outcomes, Biomarkers, Dosing Strategies)

Despite convincing preclinical data, clinical trials of oral NR have so far produced modest and often inconsistent results, particularly for metabolic endpoints. For example, in obese insulin-resistant men, a 12-week randomized, double-masked study using 2000 mg/day of NR did not improve whole-body or tissue-specific insulin sensitivity, substrate metabolism, resting energy expenditure, hepatic lipid content, or body composition; the only notable metabolic change was a small increase in plasma triglycerides, still within the normal range [68].

The same critical appraisal of 25 human NR trials highlights that the clinical literature tends to overstate positive findings, especially when they arise from small, underpowered, or non–placebo-controlled studies. In many reports, increases in MeNAM (N1-methylnicotinamide), Me2PY (N1-methyl-2-pyridone-5-carboxamide), Me4PY (N1-methyl-4-pyridone-3-carboxamide), and NAAD (Nicotinic acid adenine dinucleotide) in blood, muscle, or urine are interpreted as markers of enhanced NAD^+^ turnover. However, these metabolites may primarily reflect nicotinamide overload and disposal, rather than effective NAD^+^ repletion at the tissue level—particularly in skeletal muscle, where nicotinamide is inefficiently incorporated into NAD^+^ [65].

Several methodological issues further complicate interpretation. Trials have used a wide range of dosing regimens (from 100 to 2000 mg/day) and treatment durations, usually in small cohorts. NAD^+^ measurements have relied on assays that are not standardized across laboratories and may be unstable, and most studies report steady-state metabolite levels rather than dynamic measures of NAD^+^ flux.

Recent reviews therefore converge on a cautious view: NR reliably increases components of the NAD^+^ metabolome and may dampen inflammatory markers in selected contexts, but the evidence for clear, clinically relevant benefits remains preliminary. Larger, longer, and better-powered trials, using harmonized biomarker panels and prespecified clinical endpoints, are needed before solid therapeutic claims can be made about NR in either systemic disease or neurodegeneration [65,69].

A further conceptual limitation lies in the current biomarker repertoire. Most human studies rely on steady-state changes in circulating or urinary NAD^+^-related metabolites as proxies for tissue NAD^+^ repletion. Yet, these readouts may predominantly capture nicotinamide disposal rather than restoration of bioenergetic capacity in target organs such as skeletal muscle, heart, or brain. Incorporating dynamic flux measurements, tissue-specific imaging, or functional mitochondrial assays into future trials will be essential to disentangle genuine improvements in mitochondrial health from mere adjustments in vitamin B_3_ handling.

## 4. Berberine (BBR): Mechanisms, Preclinical and Clinical Evidence

Berberine has become one of the most thoroughly characterized examples of a “mitochondria-targeted hormetic” compound, with converging mechanistic, preclinical, and clinical data across organ systems [70,71,72,73,74].

At low concentrations it accumulates in mitochondria, where it partially inhibits complex I and perturbs oxidative phosphorylation, causing a transient rise in mitochondrial ROS, a drop in ATP with increased AMP/ATP and NAD^+^/NADH ratios, and activation of AMPK, SIRT1, NRF2 and the mitochondrial unfolded protein response; this coordinated mitohormetic program enhances antioxidant defenses, mitophagy, and cellular stress resistance, whereas higher doses shift the balance towards apoptosis and toxicity [70,71].

In the central nervous system, these same pathways translate into reduced oxidative and endoplasmic reticulum stress, attenuation of neuroinflammation, and modulation of disease-specific lesions: in multiple models of Alzheimer’s and Parkinson’s disease berberine lowers ROS, upregulates NRF2/HO-1 and SIRT1–FOXO1 signaling, promotes autophagic clearance of Aβ and hyperphosphorylated tau, and reduces caspase activation and glial activation, leading to improved cognition and motor behavior, albeit almost exclusively in preclinical studies [71].

Cardiovascular data point in a similar direction: berberine favorably remodels cardiometabolic risk by improving dyslipidemia (via LDL receptor upregulation, PCSK9 downregulation and AMPK-mediated inhibition of lipogenesis), exerting anti-atherosclerotic, anti-arrhythmic and anti-hypertensive actions, and limiting ischemia–reperfusion injury and adverse remodeling through modulation of AMPK, SIRT1, PI3K–KT–eNOS, MAPKs and PINK1/Parkin-dependent mitophagy in experimental models, with small clinical trials suggesting improvements in lipids, blood pressure, endothelial function and surrogate heart failure endpoints [72].

Importantly, these mechanistic and organ-specific effects are anchored by clinical evidence in type 2 diabetes: in a 3-month pilot trial, berberine 0.5 g t.i.d. produced HbA1c, fasting and postprandial glucose reductions comparable to metformin, while also lowering triglycerides and total cholesterol and improving HOMA-IR (Homeostasis Model Assessment of Insulin Resistance), with mainly transient gastrointestinal adverse events and no hepatic or renal toxicity [73].

A more recent meta-analysis of 37 randomized trials (3048 patients) confirms a modest but consistent glucose-lowering effect (≈0.8 mmol/L FPG, 0.6% HbA1c, 1.2 mmol/L 2-h PBG) without an increased risk of hypoglycemia or overall adverse events, and suggests that efficacy scales with baseline hyperglycemia, in line with preclinical data showing a high-glucose–dependent insulinotropic action via inhibition of pancreatic KCNH6 (Kv11.2) channels [74].

Taken together, berberine can be viewed as a prototypical low-dose mitohormetic agent with multi-system benefits in preclinical models and a growing, though still methodologically limited, clinical evidence base in metabolic and cardiometabolic disease, warranting cautious interest rather than uncritical enthusiasm [70,71,72,73,74].

## 5. Converging Mechanisms of NR and BBR: An Integrated Mechanistic Framework

### 5.1. Integrated Map of Mitochondrial Pathways Modulated by NR and BBR

**Figure 2:**
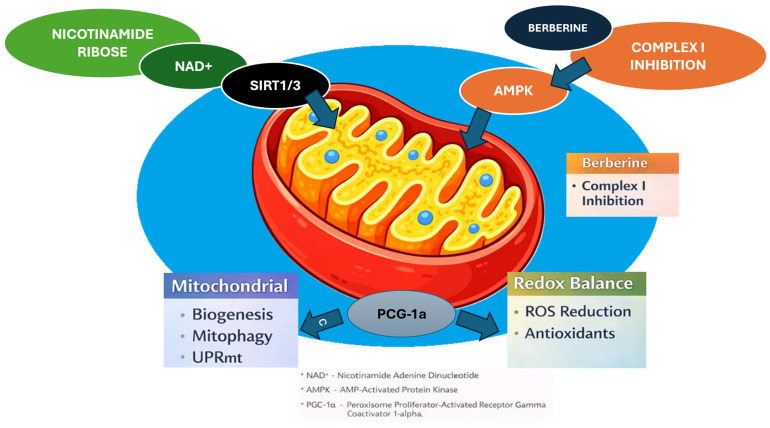
Integrated map of NR- and BBR-driven mitochondrial signaling. NR (via NAD^+^/SIRT1/3) and BBR (via complex I inhibition/AMPK) converge on PGC-1α-linked mitochondrial remodeling, promoting biogenesis/quality control and improving redox balance.

Although nicotinamide riboside (NR) and berberine (BBR) have different pathways of action, their effects converge on mitochondrial function. In particular, both act in specific signaling pathways within the mitochondria. In fact, NR primarily restores intracellular NAD+ levels, and this single change propagates through several key metabolic circuits. Increased NAD^+^ availability enhances sirtuin activity, supports mitochondrial biogenesis, and stabilizes stress-response programs that maintain organelle function under metabolic stress [24,75,76]. BBR, by contrast, reaches the mitochondrion from a different angle. Its partial inhibition of complex I produces a mild energetic strain, activating AMPK and shifting the NAD^+^/NADH ratio. This change favors adaptive remodeling, including alterations in oxidative metabolism and a gradual reinforcement of mitochondrial resilience [77,78]. When these mechanisms are combined, they outline a model where NR provides the metabolic “baseline” required to maintain a robust mitochondrial population. At the same time, BBR acts more as a trigger for acute adaptive responses. That is, NR stabilizes the system from cofactor replenishment and gene expression programs, whereas BBR pushes from below by encouraging organelles to reorganize in response to controlled metabolic stress. The final outcome, in both cases, is a more efficient and better-protected mitochondrial network. Figure 2 summarizes the shared mitochondrial signaling nodes targeted by NR and BBR and highlights their convergence on PGC-1α-linked programs governing biogenesis, quality control, and redox homeostasis.

### 5.2. Crosstalk Between AMPK, SIRT1/3, and PGC-1α in Mitochondrial Regulation

One of the most obvious points at which the two compounds interact is along the AMPK–SIRT–PGC-1α axis. By increasing NAD^+^, NR enhances SIRT1 and SIRT3 activity, driving deacetylation events that activate PGC-1α. This stimulates mitochondrial biogenesis and improves oxidative capacity, particularly in tissues experiencing chronic metabolic stress [24,75,76]. BBR hits the same regulatory system starting from AMPK. Inhibition of Complex-I spurs AMPK phosphorylation, which primes PGC-1α and, through an altered redox state, indirectly stimulates SIRT1 as well [77,78]. AMPK and SIRT1 strongly tend to potentiate each other’s effects, so that when both pathways are activated, even though different upstream triggers, cells shift toward more efficient mitochondrial metabolism and better fuel utilization [78,79,80,81]. Thus, NR and BBR don’t simply “meet” on the AMPK–SIRT–PGC-1α pathway; they reinforce opposite sides of it. NR improves cofactor-dependent pathways, while BBR energizes signaling pathways. Together, they create an ideal regulatory environment that is dynamic and responsive to metabolic changes.

### 5.3. Shared Impact on Mitophagy, UPR^mt^ and Mitochondrial Quality Control

Maintaining a healthy mitochondrial population requires continuous surveillance and, if necessary, selective removal of damaged organelles. NR directly contributes to this process by supporting PINK1/Parkin-mediated mitophagy and enhancing transcription programs that support mitochondrial chaperones and proteases. In this way, NR indirectly reduces the accumulation of dysfunctional mitochondria and the subsequent release of pro-inflammatory mitochondrial DNA fragments [24,75,76,80]. BBR, on the other hand, acts as an activator of control systems through different but partly overlapping mechanisms. In fact, by activating AMPK, BBR promotes BNIP3 and HIF-1α-dependent mitophagy and increases DRP1-mediated mitochondrial fission. This is a key step in the efficient elimination of damaged organelles [77,82]. For the UPR^mt^, NR tends to induce a broader transcriptional response—often involving the DELE1-HRI-ATF4/ATF5 axis—whereas BBR generates a more modest, hormetic pattern of activation [77]. Though differing in intensity, these responses support mitochondrial proteostasis and help to maintain a more resilient organelle network.

### 5.4. Shared Driver for Immunometabolism and Inflammation

Mitochondria are central regulators of innate immune responses, and their disruption triggers inflammatory processes via pathways including those involving cGAS–STING and the NLRP3 inflammasome. NR acts to dampen these processes by stabilizing mitochondrial membrane potential, enhancing mitophagic clearance, and reducing the presence of oxidized mtDNA in the cytosol. Enhanced sirtuin activity further reduces transcriptional priming of inflammasome-related genes [24,80]. BBR reaches a similar anti-inflammatory endpoint, though with quite different mechanisms. Improved mitochondrial redox balance and a consequent lowered ROS generation serve to reduce the release of mitochondrial DAMPs (damage-associated molecular patterns) and dampen NF-κB–dependent priming of NLRP3 (NOD-like receptor family, pyrin domain–containing 3). These actions have been reported consistently in metabolic, hepatic, and neuroinflammatory models [77,83].

Beyond direct effects on mitochondrial signaling, both NR and BBR reshape the larger inflammatory landscape. NR reduces SASP (*senescence-associated secretory phenotype*)-associated cytokines and modulates macrophage polarization through SIRT1-dependent transcriptional regulation [75,81]. BBR suppresses TLR4 (Toll-like receptor 4)/MyD88/NF-κB pathways, reduces iNOS and COX-2 (cyclooxygenase-2) expression, and favors a more anti-inflammatory cytokine profile, including higher IL-10 [77,83]. Although targeted in a differential manner, both compounds lower chronic inflammatory pressure and support the reestablishment of immune-metabolic balance.

### 5.5. Potential for Synergistic or Combinatorial Interventions

Together, these convergent mechanisms suggest that NR and BBR might complement each other in clinically meaningful ways. Thus, while NR reinforces mitochondrial capacity via NAD^+^-dependent programs, BBR provides an acute metabolic stimulus that sharpens cellular stress responses.

One plausible way to interpret the synergy between NR and BBR different benefits to view it as a stepwise process in which both compounds provide distinct stages of mitochondrial adaptation. At times, the pathway appears to begin with NR, which replenishes intracellular NAD+ levels and supports the action of sirtuin-dependent transcriptional programs. This “priming phase” prepares the metabolic environment, providing the cofactor foundation the cell needs to operate more efficiently.

Once this baseline is set, BBR seems to drive a second, more rapid layer of adaptation. AMPK activation drives the cell toward enhanced mitophagy, a more flexible redox state, and a set of rapid adaptations that protect mitochondria from energetic stress. In this respect, BBR acts almost as an acute stimulus that fine-tunes the system set by NR.

The last layer is more immunometabolic in nature. When both compounds act together, they tend to restrict the release of mitochondrial DNA, thereby reducing activation of the cGAS–STING pathway and the NLRP3 inflammasome. Such stabilization of the inflammatory environment is significant because it prevents a cascade of innate immune responses that would otherwise perpetuate mitochondrial injury [78,79,80,81,82].

## 6. Translational Applications and Disease Contexts (Human-Optimized Version)

### 6.1. Metabolic Disorders (Diabetes, NAFLD, Obesity)

Metabolic diseases such as type 2 diabetes, non-alcoholic fatty liver disease (NAFLD), and obesity all seem to share a common thread—a core pathophysiology that’s all about mitochondrial inefficiency, too much lipid buildup, and an ongoing inflammatory state. NR and BBR work in different parts of this metabolic puzzle. Still, both influence the main players in energy balance and mitochondrial health—the central regulators of how our bodies use energy. When it comes to diet-induced insulin resistance, supplementing with NR tends to boost NAD^+^ levels in tissues and improve mitochondrial function, which can enhance fatty acid burning and reduce oxidative stress [84]. But when you look at what happens in real people with established obesity or insulin resistance, NR only really starts to make a difference if they’ve got fairly mild disease so far—which suggests its benefits might be most noticeable if you catch the problem early [85,86].

On the other hand, BBR seems to have a more immediate impact thanks to its effects on AMPK. In people with NAFLD and obesity, BBR has shown up consistently in reducing liver fat, promoting the breakdown of fatty acids, and stopping the damage that lipids can cause to the mitochondria, while also helping with inflammation [87,88,89]. This makes BBR look like a pretty useful tool for getting a handle on lipid and glucose metabolism.

Putting it all together, NR seems to be more of a “get the mitochondria back in working order” kind of player by fixing the balance of some key co-factors. In contrast, BBR is more about being a strong stimulator of our body’s natural response to stress, with the two of them working together, offering a pretty good reason to think they could be useful for a range of metabolic disorders.

### 6.2. Neurodegenerative Diseases (Alzheimer’s, Parkinson’s, Ischemic Injury)

Mitochondrial decline, synaptic energetic failure, and maladaptive neuroinflammation are recurring features across neurodegenerative conditions. NAD^+^ augmentation through NR has emerged as a strategy that can simultaneously influence several of these components [90]. The NADPARK and NR-SAFE trials provided some of the clearest clinical evidence to date: oral NR reliably increases brain NAD levels in patients with early Parkinson’s disease and induces transcriptional signatures consistent with enhanced mitochondrial, lysosomal, and proteasomal activity [66,91]. It also showed that NR can stabilize mitochondrial DNA, promote the breakdown of faulty mitochondria, and reduce oxidative stress—all of which can help keep neurons alive. BBR also has neurological potential, just through different mechanisms. It engages AMPK and SIRT1, and helps reduce autophagy and inflammation in models of Parkinson’s disease and Alzheimer’s [22,92,93]. On top of all that, BBR also seems to be able to prevent microglial immune cells from becoming overactive and causing further damage.

The overlap in these mechanisms, better mitochondrial quality control, a reduced oxidative load, and an overall more manageable inflammatory response all make the two compounds a promising combination in neurodegenerative diseases.

### 6.3. Ocular Diseases (Glaucoma, Retinal Degenerations, Ischemic Retinopathy)

Given how much energy the retina and optic nerve require, the stability of mitochondria in these cells is critical. When mitochondrial function goes bad, it can lead to cell death and axonal dysfunction. Nicotinamide and NR have both shown impressive neuroprotective effects in experimental glaucoma. We’ve got multiple studies showing that taking nicotinamide orally can help keep the structure and function of retinal ganglion cells stable, and make them less vulnerable to stress [94,95]. These results are supported by reviews highlighting the relevance of NAD-based interventions in strengthening neuronal and axonal resilience [4,96].

Although BBR has not been extensively evaluated in ocular disease, its well-documented actions on mitochondrial stability, AMPK activation, and inflammatory pathways in systemic and neurological models suggest potential applicability in ischemic retinopathy and degenerative retinal conditions. Given the retina’s sensitivity to oxidative and inflammatory stress, the combined use of NR and BBR may be particularly advantageous.

### 6.4. Cardiovascular Diseases (Ischemia–Reperfusion, Heart Failure)

High levels of energy are required for normal cardiac function. In fact, cardiac tissue depends almost entirely on mitochondrial oxidative phosphorylation to perform its functions. During ischemia–reperfusion (I/R) conditions, the abrupt reintroduction of oxygen leads to multiple effects, including increased mitochondrial ROS production, destabilization of the membrane potential, and activation of apoptotic pathways. Among natural compounds, BBR has shown the most consistent cardioprotective effects. In fact, in I/R models, BBR can both preserve mitochondrial membrane potential and reduce apoptosis. By activating AMPK and reducing apoptosis, BBR limits infarct size [72,97]. Furthermore, in various studies, BBR has been shown to play an important role in post-ischemic remodeling and chronic heart failure due to its anti-inflammatory and antifibrotic properties [98]. It should also be noted that some recent studies indicate that BBR can mitigate heart failure with preserved ejection fraction by maintaining good mitochondrial homeostasis [99]. To date, direct evidence of NR in heart disease is limited. However, restoring NAD^+^ levels is increasingly recognized as a valid strategy to support redox balance and resistance to oxidative stress across various heart diseases [90].

### 6.5. Therapeutic Potential of NR + BBR Combination: Opportunities and Challenges

The idea that NR and BBR work together to improve heart health seems to be gaining strength. NR helps by promoting the growth of new mitochondria, activating certain protective pathways, and improving the heart’s energy production. Meanwhile, BBR helps by turning on a pathway that the heart uses to adapt to different situations, and which also helps remove damaged mitochondria from the heart cells. If you put the two together, it’s theoretically possible that this could give you a very powerful way to help the heart. By restoring the heart’s NAD^+^ levels, NR would provide the energy the heart needs to keep working [87,88,89]. Meanwhile, the BBR would help the heart adapt to whatever stress it’s under by turning on its own built-in protective pathways [72,97,98,99]. And because these two pathways work together and help each other out, it’s possible that putting them together could create a more efficient way for the heart to deal with damaged or failing mitochondria and overall improve the heart’s health. However, and this is a big however, there are still lots of practical challenges to overcome before this becomes a reality. For a start, we need to figure out how to make both compounds available to the cells in a way that works, and we need to understand how they interact with each other and with the heart’s energy systems. While the idea looks good on paper, we still need to conduct extensive lab and clinical research to see whether it really works in practice.

## 7. Challenges and Future Perspectives

### 7.1. Methodological Limitations of Current Clinical Trials

Despite growing enthusiasm for NR and BBR as modulators of mitochondrial biology, clinical evidence remains limited due to several methodological issues. Most studies of NR so far have had small patient cohorts and short treatment durations. In these cases, statistical power is limited, making it difficult to detect even subtle metabolic or mitochondrial changes [100,101,102]. In addition, variability in the administered dosage makes it difficult to reconcile and unify results across different studies. Many studies in the literature have also included patients with chronic metabolic or neurodegenerative diseases, in whom mitochondrial damage may be too important and irreversible to respond to a possible restoration of NAD^+^. BBR clinical trials, on the other hand, present a different type of problem. The low and variable bioavailability of the compound determines a non-negligible variability in studies [103,104]. In addition, it should be noted that many studies have used indirect metabolic measures rather than direct measures of mitochondrial function. Overall, however, these limitations highlight the need for studies designed with validated mitochondrial endpoints and standardized compound formulations.

### 7.2. Need for Reliable Biomarkers of Mitochondrial Function (NAD^+^ Levels, Extracellular Vesicles, Multi-Omics)

A major obstacle in the evaluation of NR and BBR in clinical practice is the lack of reliable biomarkers of mitochondrial function in vivo. Peripheral NAD^+^ measurements, while widely used, offer only a fragmented view of the cellular redox landscape and do not necessarily reflect mitochondrial NAD^+^ pools [30]. New analytical strategies are needed. New emerging approaches could help overcome these obstacles; for example, profiling mitochondrial burden in extracellular vesicles or quantifying circulating mtDNA signatures [105]. In addition, multi-omics pictures combining transcribed, proteomic, lipidomic, and metabolomic data are proving reliable in assessing mitochondrial adaptations over time [106]. Standardized protocols and validation between different cohorts will be essential before these biomarkers can be used in next-generation clinical trials.

### 7.3. Advances in Pharmacokinetics and Formulation Strategies (e.g., NRH, BBR Derivatives)

Rapid advancement in mitochondrial pharmacology has led to the development of more potent and bioavailable NRs and BBRs. Among the most exciting is NRH, a reduced precursor to NAD^+^ that is more stable, more readily taken up by cells, and much more potent at increasing intracellular NAD^+^ levels than classical NR [107]. Improving the pharmacokinetic profile of NRH (reduced nicotinamide riboside) opens up therapeutic strategies that were not possible with NR alone. Meanwhile, significant efforts have been devoted to overcoming the poor oral bioavailability of BBR. New formulations—including nanoencapsulated constructs, phospholipid complexes, saline derivatives and chemically modified analogues—have produced compounds with improved absorption, increased tissue penetration and, in some cases, reduced gastrointestinal side effects [103,108]. Some of these next-generation BBR derivatives exhibit improved AMPK activation, so more consistent mitochondrial involvement may be possible with optimized formulations.

### 7.4. Risk Assessment, Safety, and Potential Adverse Interactions

NR and BBR are generally considered safe molecules. However, even in this case, it is necessary to monitor their safety profile over time during their clinical use. In fact, too high doses of NR increase circulating levels of nicotinamide and its metabolites. These on predisposed individuals could exert additional stress on mitochondrial metabolism or negatively affect sirtuin-dependent signaling pathways [50]. The long-term consequences of chronic elevation of NAD^+^ levels have not been fully characterized and require evaluation. The safety profile of BBR has been established, but the compound interacts with metabolizer enzymes and drug transporters, so there are potential concerns in patients taking multiple drugs. BBR can affect CYP450 isoforms and P-glycoprotein, thereby altering the pharmacokinetics of commonly prescribed drugs such as statins, metformin, and anticoagulants [109,110]. When considering NR–BBR combination therapy, additional uncertainties emerge as chronic modulation of both NAD^+^/NADH and AMP/ATP ratios could produce tissue-specific effects that have not been experimentally tested. Comprehensive drug–drug interaction studies and long-term toxicological evaluations will be needed before combination regimens can be tested in large patient populations.

## 8. Conclusions

Mitochondrial dysfunction is a common thread between metabolic, neurodegenerative, ocular, and cardiovascular diseases. In this context, nicotinamide riboside (NR) and berberine (BBR) are two mechanistically different but functionally convergent modulators of mitochondrial homeostasis. During this review, we demonstrated that NR works by restoring the intracellular pool of NAD^+^ and sirtuin-driven programs of mitochondrial biogenesis, genomic maintenance, and metabolic resilience. BBR acts via mild energy stress and AMPK activation, triggering adaptive remodeling of mitochondrial dynamics, mitophagy, and attenuation of inflammatory responses. Although their molecular “entry points” differ, NR and BBR intersect at several critical nodes—PGC-1α activation, mitochondrial quality-control pathways, and immunometabolic regulation. This convergence provides a strong mechanistic justification for combining them, especially in diseases in which mitochondrial failure and chronic inflammation reinforce one another. However, many challenges lie ahead before these compounds can be translated into clinical practice. Current clinical trials are limited by small sample sizes, short heterogeneous durations and targets, and the absence of in vivo biomarkers of mitochondrial function. In addition, the pharmacokinetic constraints of both NR and BBR, as well as uncertainty about long-term safety, metabolic interactions, and tissue-specific responses, must be considered. Advances in biomarker development, next-generation NAD^+^ precursors such as NRH, and bioavailability-enhanced BBR derivatives could help overcome these barriers. Ultimately, the therapeutic potential of NR, BBR and their combination lies not in correcting a single metabolic defect, but in restoring mitochondrial integrity across multiple layers of cellular physiology. Addressing methodological, pharmacological, and mechanistic gaps in this review will be essential to define its translational value. Future research integrating multi-omics profiling, system-wide mitochondrial biomarkers, and rigorously designed combined studies will tell us whether these compounds can go from being promising mitochondrial modulators to clinically meaningful therapies.

## Data Availability

No new data were created or analyzed in this study.

## Abbreviations

AAV2, adeno-associated virus serotype 2; AD, Alzheimer’s disease; ADOA, Autosomal dominant optic atrophy; AIM2, absent in melanoma 2; ALS, amyotrophic lateral sclerosis; AMBRA1, autophagy and beclin 1 regulator 1; AMPK, AMP-activated protein kinase; ATP, adenosine triphosphate; ATF4, activating transcription factor 4; ATF5, activating transcription factor 5; BAK, BCL2 antagonist/killer 1; BAX, BCL2-associated X protein; BBR, berberine; BBB, blood–brain barrier; BNIP3, BCL2 interacting protein 3; cGAS, cyclic GMP-AMP synthase; CHOP, C/EBP homologous protein; CI–CIV, mitochondrial respiratory chain complexes I–IV; CoQ, coenzyme Q (ubiquinone); CoQ10, coenzyme Q10; COX-2, cyclooxygenase-2; CNS, central nervous system; Cyt c, cytochrome c; DAMP, damage-associated molecular pattern; DRP1, dynamin-related protein 1; eIF2α, eukaryotic initiation factor 2 alpha; eNAMPT, extracellular nicotinamide phosphoribosyltransferase; ERR, estrogen-related receptor; FPG, fasting plasma glucose; FUNDC1, FUN14 domain containing 1; G6Pase, glucose-6-phosphatase; GFAP, glial fibrillary acidic protein; HbA1c, glycated hemoglobin; HIF-1α, hypoxia-inducible factor 1 alpha; HO-1, heme oxygenase-1; HOMA-IR, homeostatic model assessment of insulin resistance; HRI, heme-regulated inhibitor kinase; HK2, hexokinase 2; IL, interleukin; iNOS, inducible nitric oxide synthase; IOP, intraocular pressure; I/R, ischemia–reperfusion; LC3, microtubule-associated protein 1A/1B-light chain 3; LED, light-emitting diode; LHON, Leber hereditary optic neuropathy; MAPK, mitogen-activated protein kinase; Me2PY, N-methyl-2-pyridone-5-carboxamide; Me4PY, N-methyl-4-pyridone-3-carboxamide; MeNAM, N-methylnicotinamide; MFN1/2, mitofusin 1/2; mPTP, mitochondrial permeability transition pore; MRC, mitochondrial respiratory chain; mTOR, mechanistic target of rapamycin; mtDNA, mitochondrial DNA; mtROS, mitochondrial reactive oxygen species; MOMP, mitochondrial outer membrane permeabilization; MYOC, myocilin; NAAD, nicotinic acid adenine dinucleotide; NAD^+^, nicotinamide adenine dinucleotide; NAFLD, non-alcoholic fatty liver disease; NAM, nicotinamide; NAMPT, nicotinamide phosphoribosyltransferase; ND4, NADH dehydrogenase subunit 4; NF-κB, nuclear factor kappa B; NIX (BNIP3L), NIP3-like protein X; NLRP3, NLR family pyrin domain-containing 3; NMN, nicotinamide mononucleotide; NMNAT, nicotinamide mononucleotide adenylyltransferase; NOX, NADPH oxidase; Nrf2, nuclear factor erythroid 2-related factor 2; NR, nicotinamide riboside; NRF1/2, nuclear respiratory factor 1/2; NRK, nicotinamide riboside kinase; NTG, normal-tension glaucoma; OAG, open-angle glaucoma; OMA1, OMA1 zinc metallopeptidase; ONH, optic nerve head; OPA1, optic atrophy 1; OXPHOS, oxidative phosphorylation; PARP, poly(ADP-ribose) polymerase; PBG, postprandial blood glucose; PCSK9, proprotein convertase subtilisin/kexin type 9; PEPCK, phosphoenolpyruvate carboxykinase; PGC-1α, peroxisome proliferator-activated receptor gamma coactivator 1-alpha; PI3K, phosphoinositide 3-kinase; PINK1, PTEN-induced kinase 1; POAG, primary open-angle glaucoma; PRPP, phosphoribosyl pyrophosphate; RGC, retinal ganglion cell; ROCK1, Rho-associated coiled-coil-containing protein kinase 1; ROS, reactive oxygen species; SASP, senescence-associated secretory phenotype; SARM1, sterile alpha and TIR motif-containing 1; SC, supercomplex; SIRT, sirtuin; SkQ1, plastoquinonyl-decyltriphenylphosphonium; SOD2, superoxide dismutase 2; SS-31, Szeto–Schiller peptide 31 (elamipretide); STING, stimulator of interferon genes; TBK1, TANK-binding kinase 1; TFAM, mitochondrial transcription factor A; TLR, Toll-like receptor; TLR4, Toll-like receptor 4; TLR9, Toll-like receptor 9; TNF-α, tumor necrosis factor alpha; TP53, tumor protein p53; UCP1/2, uncoupling protein 1/2; UPR^mt^, mitochondrial unfolded protein response; VDAC, voltage-dependent anion channel.
